# Bone marrow mesenchymal stromal cells from acute myelogenous leukemia patients demonstrate adipogenic differentiation propensity with implications for leukemia cell support

**DOI:** 10.1038/s41375-019-0568-8

**Published:** 2019-09-06

**Authors:** Mitra Azadniv, Jason R. Myers, Helene R. McMurray, Naxin Guo, Phil Rock, Myra L. Coppage, John Ashton, Michael W. Becker, Laura M. Calvi, Jane L. Liesveld

**Affiliations:** 1 Department of Medicine, Hematology/Oncology, University of Rochester, Rochester, NY USA; 2 The James P Wilmot Cancer Institute, University of Rochester, Rochester, NY USA; 3 Genomics Research Center, University of Rochester, Rochester, NY USA; 4 Pathology and Laboratory Medicine, University of Rochester, Rochester, NY USA; 5 Department of Medicine, Division of Endocrinology, University of Rochester, Rochester, NY USA

**Keywords:** Acute myeloid leukaemia, Cancer microenvironment

## Abstract

Bone marrow mesenchymal stromal cells (MSCs) constitute one of the important components of the hematopoietic microenvironmental niche. In vivo studies have shown that depletion of marrow MSCs resulted in reduction of hematopoietic stem cell content, and there is in vitro evidence that marrow MSCs are able to support leukemia progenitor cell proliferation and survival and provide resistance to cytotoxic therapies. How MSCs from leukemia marrow differ from normal counterparts and how they are influenced by the presence of leukemia stem and progenitor cells are still incompletely understood. In this work, we compared normal donor (ND) and acute myelogenous leukemia (AML) derived MSCs and found that AML-MSCs had increased adipogenic potential with improved ability to support survival of leukemia progenitor cells. To identify underlying changes, RNA-Seq analysis was performed. Gene ontology and pathway analysis revealed adipogenesis to be among the set of altered biological pathways dysregulated in AML-MSCs as compared with ND-MSCs. Expression of both *SOX9* and *EGR2* was decreased in AML-MSCs as compared with ND-MSCs. Increasing expression of SOX9 decreased adipogenic potential of AML-MSCs and decreased their ability to support AML progenitor cells. These findings suggest that AML-MSCs possess adipogenic potential which may enhance support of leukemia progenitor cells.

## Introduction

Acute myelogenous leukemia (AML) is characterized by proliferation and/or accumulation of abnormal clone(s) of cells which lack differentiation potential. Concurrently, production of normal hematopoietic cells is curtailed, often resulting in clinically significant cytopenias. There were an estimated 21,380 new cases of AML diagnosed in the United States in 2017 [1]. AML is a heterogeneous disease with multiple subtypes [2, 3], and survival of AML patients is poor at present as about 30% of young adults and 90% of older adults die of their illness [4]. Complete remission rates are high, but relapse often occurs. Relapse is thought to arise from undetectable leukemia cells which survive in the marrow microenvironment after treatment. Several studies in murine models and with human marrow ex vivo have suggested that the bone marrow microenvironment plays an important function in supporting the survival of leukemia stem cells, presumably contributing to therapeutic resistance and disease relapse [5, 6].

The marrow microenvironment includes a complex network of extracellular matrix proteins, soluble growth factors, cytokines, and distinct but possibly overlapping cellular niches, including the endosteal (or osteoblastic) niche, and the vascular niche [7]. While an oversimplification, osteoblasts and mesenchymal progenitor cells along with endosteal fibroblasts compromise the endosteal niche, and the vascular niche is defined by its proximity to vascular endothelial cells [8, 9]. Adipocytes are an important contributor to the marrow environment as well. Bone marrow adipocytes generally correlate inversely with the hematopoietic activity of the marrow. In mice, for example, HSCs and short-term progenitors are reduced in frequency in the vertebrae of the tail where adipocytes predominate vs. in thoracic vertebrae where they are less prominent [10].

In human marrow, the definition of distinct niches and hierarchy of mesenchymal stromal cells (MSCs) is less well-characterized than in murine models. In murine models, MSCs have been classified as CXCL12-abundant reticular cells, stem cell factor expressing cells, nestin-expressing cells, and PDGFR-α, Sca-1^+^, CD45^−^, and Ter119^−^ cells [11]. In humans, MSCs must be adherent to plastic, have multipotent differentiation capability, and express CD73, CD90, and CD105 [12]. Marrow MSCs are reported to have an important role in regulating hematopoiesis under physiological conditions [13]. In leukemia states, there is evidence that marrow niches are altered to support leukemogenesis and to disrupt normal hematopoiesis [14–17]. Several studies have indicated MSCs can provide survival and antiapoptotic signals to AML cells and as a result protect them from chemotherapy [5]. To what extent MSCs are altered in AML and to what extent they contribute to altered niche effects are underexplored.

There is experimental evidence in murine models that changes in microenvironmental cells can result in myeloproliferative disease through Notch activation [18], in myelodysplasia (MDS) through DICER knockout [19], and in leukemia through activating mutations of beta-catenin [20]. MSCs in AML may have cytogenetic aberrations, although distinct from of those of the leukemic clone, suggesting that the developing leukemia may enable alterations to accumulate in MSCs [21]. Other studies in MDS have also shown that exposure to dysplastic cells reprograms MSCs to establish a distinct stem cell niche [22], and there is some evidence for this in AML as well [15].

There are now several reports of MSC changes in human hematologic malignancies such as MDS [23, 24], acute lymphoblastic leukemia [25], and AML [14, 21, 26–28]. Some of these reports suggest increased osteogenic potential [14] whereas others have shown increased adipogenic potential [26, 29]. While the osteoblast contribution to the niche has received much attention, adipocyte presence is also thought to influence leukemia cells and their responses to chemotherapeutic agents [28, 30, 31]. Despite the heterogeneity of AML, MSC heterogeneity has been found by some investigators to be minimal across subtypes [32, 33], suggesting that MSCs could be a universal target in the therapy of AML despite its heterogeneity.

In this work we sought to further compare ND-MSCs and AML-MSCs by phenotype and transcriptome analysis in order to assess effect on outgrowth of leukemia blasts and progenitors. Our findings, using primary marrow from AML patients, show an adipogenic predisposition in MSCs isolated from established AML and suggest that these changes may impact leukemia cell survival in the marrow niche.

## Materials and methods

Please refer to Supplementary Information for more detailed “Materials and methods” descriptions.

### Characteristics of clinical samples

MSCs were cultured from marrow of 11 AML patients, 8 previously untreated (Supplementary Table 1) who gave written consent for use of marrow aspirates for research purposes or from de-identified AML specimens as approved by the Research Subjects Review Board at the University of Rochester. ND-MSCs were aspirated from normal subjects with written informed consent (*n* = 10; Supplementary Table 1) or were grown from de-identified specimens from marrows obtained during lymphoma staging which were free of disease (*n* = 1; Supplementary Table 1). AML-MSCs were all from M1, M2, or M4 FAB (French/American/British) subtypes. In keeping with median age distribution of AML, all normal donor (ND), and AML specimens were from subjects >50 years old. To obviate stromal cell contamination, AML blast specimens used for co-culture with MSCs were from cryopreserved apheresis samples which were generally of M4 or M5 subtype with >80% blasts at diagnosis. Only specimens from untreated patients were used in transcription and co-culture experiments.

### MSC differentiation

ND- and AML-MSCs were differentiated along osteogenic, adipogenic, and chondrogenic pathways using standard methodologies as described in Supplementary Materials. All MSC monolayers were at passage 3 at the start of differentiation, and all control monolayers were utilized at passage 3 as well.

### RNA-Seq and pathway analysis

All RNA-Seq data were processed on Illumina HiSeq2500v4. Cleaned read data for each sample were aligned to the human genome (hg38) with STAR-2.5.2b. The list of significant genes was used for pathway analysis using Ingenuity Pathway Analysis software (IPA; Qiagen Bioinformatics; Redwood City, CA, USA).

### Enhancement of SOX9 expression

SOX9 lentiviral activation particles were utilized following manufacturer’s instructions (sc-400143-LAC; Santa Cruz Biotechnology, Dallas, TX, USA) to upregulate expression of the SOX9 gene. This is a synergistic activation mediator (SAM) transcription activation system. In negative controls, lentiviral activation particles contain SAM activation elements which are deactivated via Cas9 nuclease fused to the transactivation domain and a nonspecific 20 nucleotide guide RNA. The SAM complex does not recognize or bind any specific sequence within the genomic DNA and does not activate transcription of any specific gene.

### Statistical analysis

All data were collected from a minimum of three independent experiments. Data were expressed as the mean ± standard error of means from the indicated number of experiments. Comparisons among groups were conducted using one-way analysis of variance. Student’s *t* test was used to test differences between groups. Statistical significance was considered at *p* < 0.05 utilizing prism (Graphpad, La Jolla, CA, USA).

## Results

### Morphologic and phenotypic characteristics of ND-MSCs vs. AML-MSCs

**Fig. 1:**
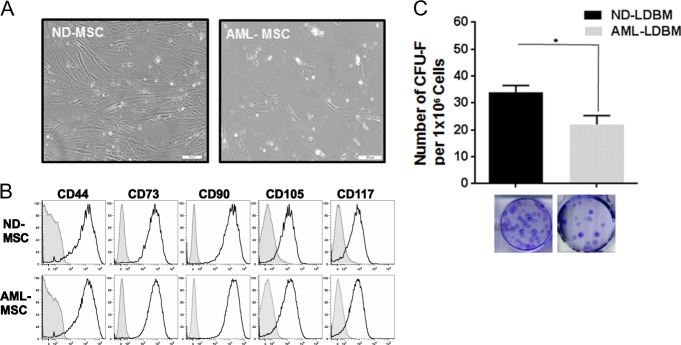
Morphological characteristics, surface antigen expression and colony forming unit (CFU-F) potential of bone marrow-derived mesenchymal stem cells. **a** Phase-contrast images of MSCs from a normal donor (ND) having similar shape as compared with MSCs derived from an AML patient. Both ND- and AML-MSCs are at passage 1 and scale bar is 50 μm. **b** Representative flow cytometry profiles of surface marker expression on ND and AML-MSCs. **c** The CFU-F potential of AML-LDBM vs. ND-LDBM; (*p* = 0.0002; *n* = 5). Representative images of ND and AML CFU-Fs are shown in the bottom panel

ND-MSCs had similar morphology as compared with age-matched AML-MSCs as presented in Fig. 1a, and in general these cells had irregular shape and evidence for branching. To further characterize the MSCs, they were immunophenotyped via flow cytometry analysis of surface antigen expression. This revealed positive expression of CD44, CD73, CD90, CD105, and CD117 (Fig. 1b). There were no significant differences in the percent expression of these MSC defining markers between ND-MSCs and AML-MSCs (Supplementary Fig. 1A1). The gating strategy for flow cytometry is shown in Supplementary Fig. 1A2. Both AML and ND-MSCs expressed CD106, CD146, and CD271 and were negative for CD31, CD14, and CD45 (Data not shown). There was no significant difference in the mean fluorescence intensity of CD106, CD146, and CD271 between ND-MSCs and AML-MSCs (Data not shown). There were no statistically significant differences in the rates of apoptosis and senescence between ND-MSCs and AML-MSCs as measured by Annexin V and β-galactosidase expression, respectively (Supplementary Fig. 1B and 1 C; *p* > 0.05, *n* = 5).

### Time to confluence of ND-MSCs vs. AML-MSCs

Light density bone marrow (LDBM) cells were plated at a density of 5 × 10^5^/cm^2^ in tissue culture flasks for both ND and AML specimens. Early passaged AML-MSCs lagged behind in their growth rate as defined by days between passage to confluence as compared with ND-MSCs (*p = 0.0058, **p = 0.00013) but this difference was not observed in later passaged cells (Supplementary Fig. 1D).

### Colony forming unit (CFU)-F potential of normal vs. AML-LDBM cells

The CFU-F assay was performed using LDBM from ND and AML samples in order to evaluate their fibroblastic clonogenic potential. The CFU-F outgrowth was significantly decreased in AML-LDBM as compared with that from age-matched normal marrow donors (Fig. 1c). In the NDs the mean CFU-F was 34 as compared with 21 in the AML samples (*p = 0.0002, *n* = 5) per 1 million LDBM cells plated. Representative images of CFU-F from ND and AML-LDBM are shown in the lower panel of Fig. 1c.

### Osteogenic differentiation potential of ND-MSCs vs. AML-MSCs

**Fig. 2:**
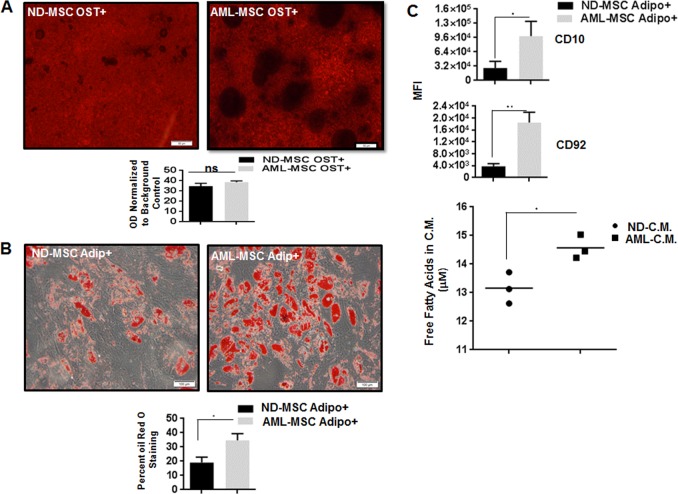
Differentiation potential of ND-MSCs vs. AML-MSCs. **a** ND-MSCs have the same osteogenic differentiation ability as AML-MSCs. Representative images of ND-MSCs and AML-MSCs following two rounds of osteogenic induction (Ost+) (scale bar = 50 μm). Alizarin red staining (lower panel of A) showed no difference between ND-MSCs and AML-MSCs (*p* = 0.35; *n* = 4). **b** Staining with oil Red O showed lower lipid droplet staining in ND-MSCs as compared with that in AML-MSCs after adipogenic induction (Adipo+). Percent of MSCs stained with oil Red O was determined in ND-MSCs vs. AML-MSCs (*p = 0.0015; *n* = 5). **c** CD10 and CD92 expression were determined on ND-MSCs and AML-MSCs after two rounds of induction to adipocytes. The CD10 and CD92 mean fluorescent intensity (MFI) was increased in AML vs. ND-MSCs (*p = 0.04 and **p = 0.0032; *n* = 4). **c** The level of free fatty acid released into the conditioned medium (C.M.) collected from cultured noninduced ND and AML-MSCs was measured (*p = 0.024; *n* = 3)

After two rounds of exposure to osteogenic induction medium, no difference was noted between ND- and AML-derived MSCs. This was measured by Alizarin red S staining (Fig. 2a) and by assessment of mineralization through acetic acid extraction and neutralization with ammonium hydroxide followed by colorimetric detection at 405 nm (Fig. 2a; *p* = 0.35, *n* = 4).

### Adipogenic differentiation potential of ND-MSCs vs. AML-MSCs

After two rounds of induction in adipogenic medium at passage 3, AML-MSCs exhibited more oil Red O stained cells as compared with ND-MSCs from age-matched controls also at passage 3 (Fig. 2b). The percent oil Red O stained AML-MSCs and ND- MSCs was determined by a blinded observer (Fig. 2b). Confirming the adipogenic features of AML-MSCs vs. ND-MSCs, the AML-MSCs expressed more CD10 and CD92 than did their normal counterparts (Fig. 2c). CD10 is a surface marker metalloendopeptidase enzyme expressed on some lymphoid cells and MSCs. Expression of CD10 has been reported to be upregulated during adipogenic differentiation [34]. CD92, a 70kd choline transporter important for acetylcholine synthesis, has also been found to be increased in adipogenic MSCs [34]. MSCs from AML samples were also found to secrete more free fatty acid into conditioned medium than their normal counterparts as quantified by ELISA (Fig. 2c, lower panel).

### Identification of a unique gene signature in ND-MSCs vs. AML-MSCs

**Fig. 3:**
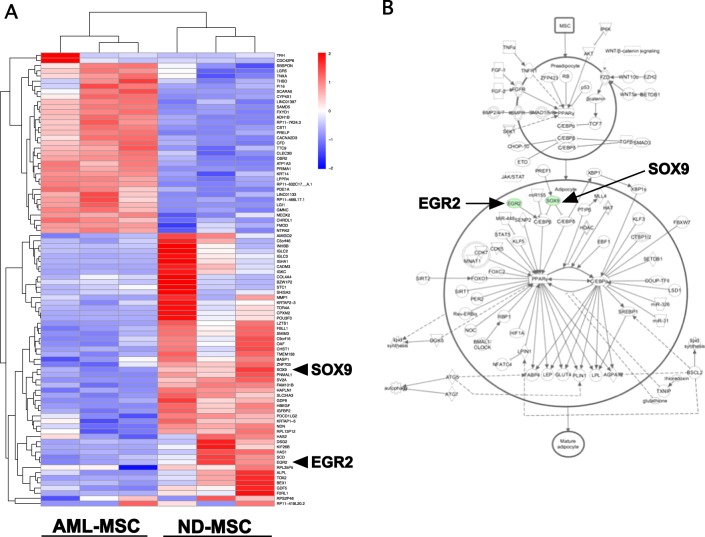
Heat map of differentially expressed genes between ND-MSCs and AML-MSCs. **a** Hierarchically clustered heat map of mRNA expression for 88 significantly differentially expressed genes (CuffDiff *q* value < 0.05). Color key represents raw scaling of the log(FPKM + 1) expression values. **b** Adipogenesis pathway analysis related to differentially expressed genes. Ingenuity pathway analysis (IPA) proposed a model of altered fatty acid expression and adipogenic differentiation in AML-MSCs attributable to lower expression of two key regulatory genes, *SOX9* and *EGR2* shown in green and highlighted by arrows

In order to assess differences in gene expression between ND-MSCs and AML-MSCs, an unbiased RNA-Seq analysis was performed on three ND-MSC and three previously untreated AML-MSC specimens, all from subjects >50 years of age and analyzed at passage 3 in culture. This identified 88 genes significantly differentially expressed between the two groups (*q* < 0.05). Lists of up- and downregulated genes are shown in Supplementary Tables 2 and 3, respectively. A heat map was generated showing raw-scaled log(FPKM (fragments per kilobase of exon model per million reads mapped) + 1) expression values. This readily grouped samples by type (ND vs. AML, Fig. 3a).

### Pathway analysis of differentially expressed genes between ND-MSCs and AML-MSCs

Analysis using IPA was conducted based on log2-fold changes in gene expression. This predicted dysregulation of four canonical pathways in AML-MSCs as compared with ND-MSCs (Supplementary Fig. 2). These included role of tissue factor in cancer, airway pathology, oleate biosynthesis, and adipogenesis. IPA analysis proposed a model of altered adipogenic differentiation in AML-MSCs attributable to lower expression of two key regulatory genes, *SOX9* (SRY-related high mobility group-Box gene 9) and *EGR2* (early growth response gene 2; Fig. 3b).

### qRT-PCR and western blot validation of SOX9 and EGR2 expression changes

**Fig. 4:**
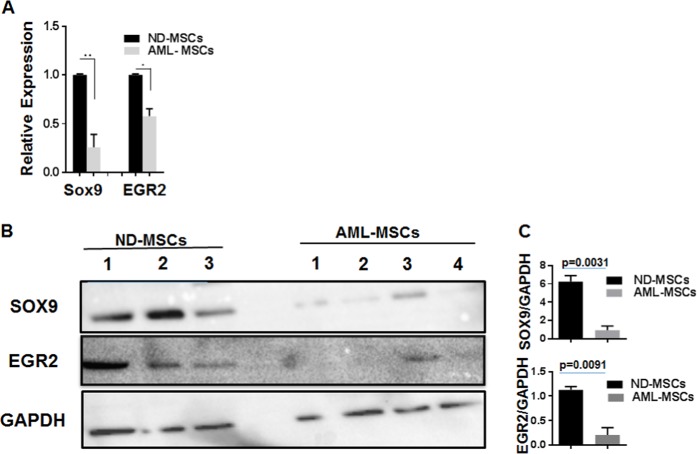
qRT-PCR and western blot of SOX9 and EGR2 expression. **a** Reduced expression of SOX9 and EGR2 in AML-MSCs as compared with ND-MSCs was validated by qRT-PCR (*p = 0.0014, **p < 0.0001; *n* = 6). **b** Western blot demonstrated decreased levels of SOX9 and EGR2 expression in AML-MSCs (*n* = 4) vs. ND-MSCs (*n* = 3). **c** Mean band densities from western blot in B normalized relative to GAPDH for SOX9 and EGR2

Reduced expression of SOX9 and EGR2 in AML-MSCs as compared with ND-MSCs was validated by qRT-PCR (Fig. 4a) and western blot analysis (Fig. 4b, c). There was some variation in the expression in AML-MSC samples. Despite no significant differences in gene expression by RNA-Seq, increased gene expression of fatty acid binding protein 4 (FABP4) was demonstrated by qRT-PCR in AML-MSCs as compared with ND-MSCs (Supplementary Fig. 3). However, no significant difference was detected in the expression of PPARγ (peroxisome proliferator-activated receptor gamma), although a trend toward increased expression in AML-MSCs was noted.

### Effect of AML-MSCs and ND-MSCs on leukemia blast and progenitor survival

**Fig. 5:**
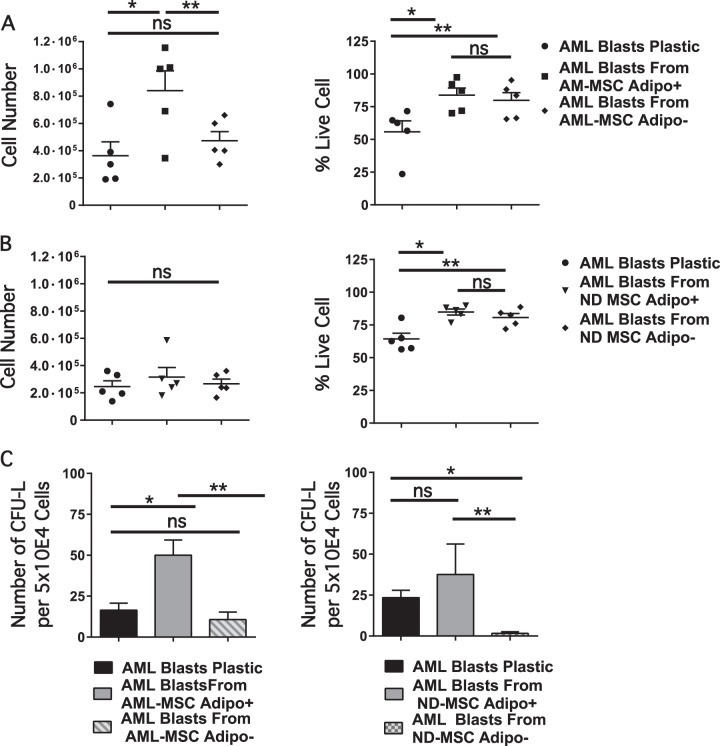
Annexin V expression and percentage live AML blasts co-cultured on AML-MSCs and ND-MSCs induced and noninduced in adipogenic media. **a** Cell number (left panel, *p = 0.0138, **p = 0.025, *n* = 5) and percent live AML blast cells (right panel, *p = 0.0093, **p = 0.007; *n* = 5) cultured over AML-MSCs induced to adipocytes or uninduced as compared with blasts cultured by themselves for 72 h. **b** Cell number and percent live AML blasts from induced or uninduced ND-MSC as compared with AML blasts in tissue culture plastic control (Left panel, ns = not significant; *n* = 5; right panel, *p = 0.002, **p = 0.001; *n* = 5). **c** Top panel. CFU-L outgrowth after co-culture with AML-MSCs adipogenic induced, uninduced, or over tissue culture plastic control (*p = 0.0008, **p = 0.0003, ns = not significant). Bottom panel. Induced or uninduced ND-MSC support of CFU-Ls as compared with co-culture over tissue culture plastic control (ns = not significant, *p = 0.0002, **p = 0.033; *n* = 5). In these experiments, five distinct AML-MSCs or ND-MSCs monolayers were utilized; three co-cultured with one AML apheresis sample and two cultured with another AML apheresis sample. Adipo− = uninduced MSC layer; Adipo+ = MSC layer induced with two rounds of induction media

Survival of primary blasts co-cultured with MSCs from five distinct AML cases was significantly greater than that of AML blasts cultured alone for 72 h. Representative flow analysis plots are shown in Supplementary Fig. 4A. After 3 days of co-culture, no significant early or late apoptosis was noted in any culture condition. AML blast cells cultured by themselves for 3 days had a significant decrease in the total cell number and percent of live cells as compared with primary AML blasts cultured on adipocyte induced AML-MSCs (Fig. 5a; left panel). However, the percentage of live AML blast cells from AML-MSCs induced to adipocytes vs. noninduced was not statistically different (Fig. 5a; right panel). There were no differences in total cell number from co-culture of primary blasts over plastic as compared with adipogenic induced or uninduced ND-MSCs (Fig. 5b; Left panel). The percentage of live cells was greater over both induced and uninduced ND-MSCs monolayers (Fig. 5b; right panel). To see if apoptosis could be demonstrated at earlier time points, after 24 h of co-culture in serum free media, the percentage of late apoptotic cells was significantly higher in AML blasts cultured on plastic than on either ND or AML-MSCs noninduced or induced to adipogenesis (Supplementary Fig. 4B).

To determine whether there was differential capability between AML-MSCs and ND-MSCs either induced or noninduced to adipogenesis to support clonogenic progenitors, CFU-L assays were performed with primary AML blasts removed from co-culture assays. The AML-MSCs induced to adipocytes supported a higher number of CFU-L per equivalent number of cells plated as compared with AML blast cells in tissue culture plastic or over uninduced MSCs (Fig. 5c). When primary AML blasts were co-cultured with ND-MSCs, there was no significant difference in CFU-L outgrowth as compared with outgrowth from primary blasts in culture medium alone (Fig. 5c; Lower plot).

### Effect of ND-MSCs and AML-MSCs on CFU-GM and BFU-E outgrowth

**Fig. 6:**
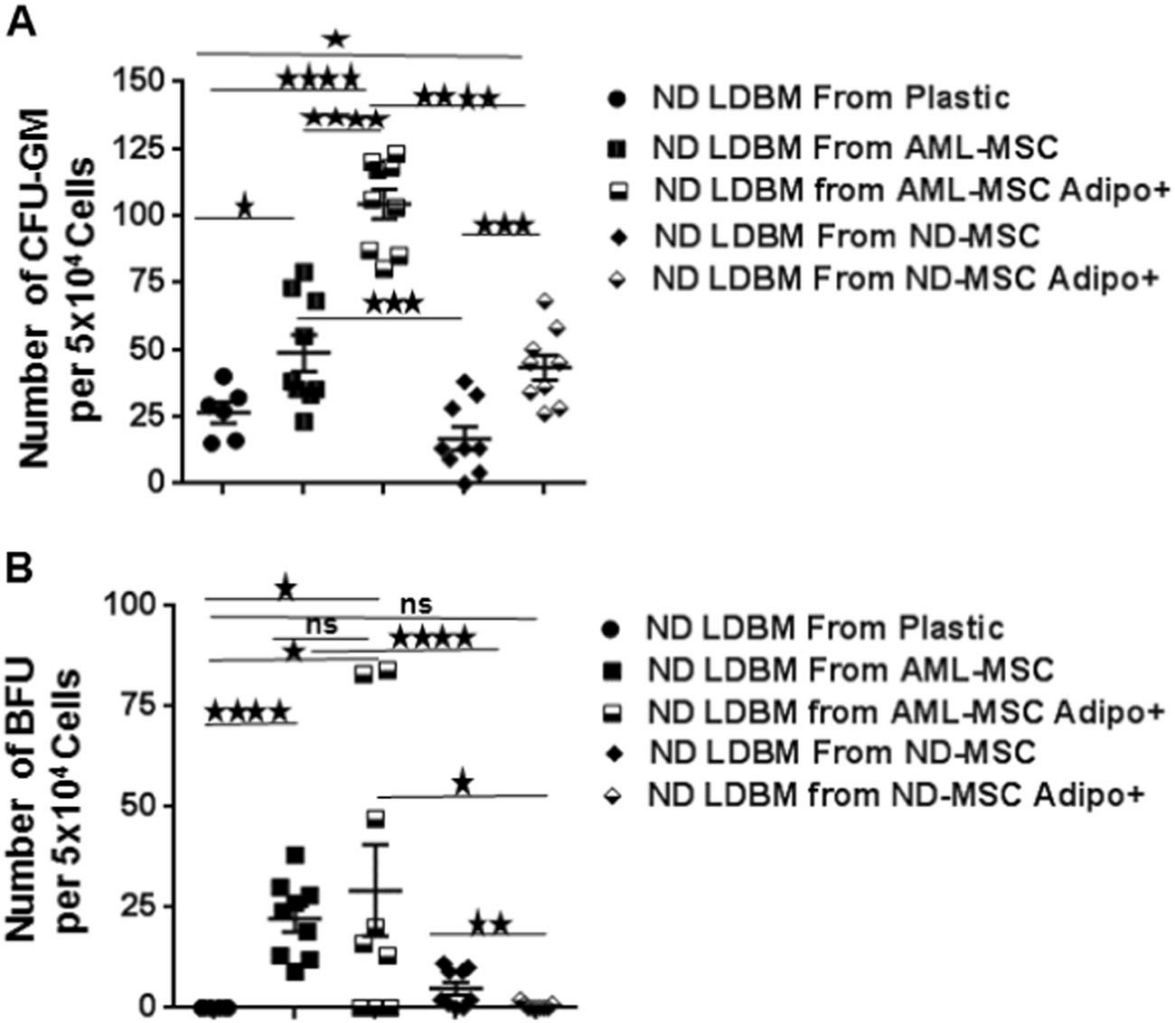
AML-MSC support of CFU-GM and BFU-E colonies as compared with ND-MSCs. ND-LDBM cells from the same subject were co-cultured for 72 h with indicated MSCs or on plastic and then plated for CFU-GM and BFU-E outgrowth. Three different ND and AML-MSCs were used in these experiments. **a** CFU-GM (*p = 0.0138, ***p = 0.0004, ****p ≤ 0.0001), and **b** BFU-E support by AML-MSCs or ND-MSCs (*p < 0.05, **p = 0.0064, ***p = 0.0004, ****p < 0.0001, ns = not significant)

To determine whether there was differential ability between AML and ND-MSCs to support normal clonogenic progenitors, CFU-GM and BFU-E assays were conducted with LDBM cells from a normal subject cultured over three distinct AML and ND-MSC layers. AML-MSCs supported higher numbers of both CFU-GMs (Fig. 6a) and BFU-Es (Fig. 6b) as compared with culture of normal LDBM over tissue culture plastic or co-culture on ND-MSCs for 3 days. Adipocyte induction of ND- and AML-MSCs resulted in better support of CFU-GMs as compared with uninduced ND and AML-MSCs (Fig. 6a). AML-MSCs induced to adipocytes had better support for CFU-GMs as compared with ND-MSCs induced to adipocytes. There was no difference in BFU-E output between baseline and adipocyte-induced MSCs, but BFU-E output appeared better over AML-MSCs (Fig. 6b).

### Enhancement of SOX9 gene expression

**Fig. 7:**
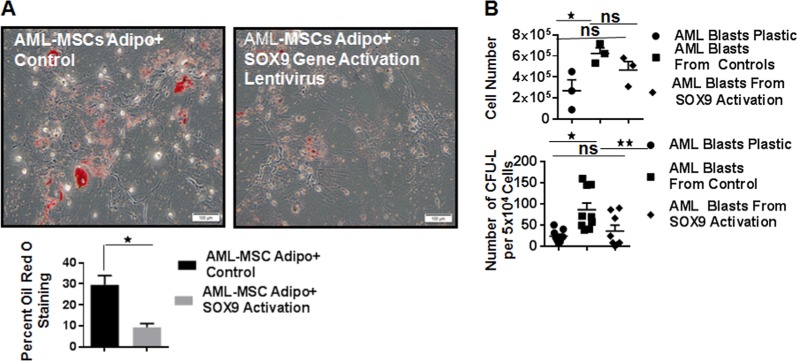
Effect of enhancement of SOX9 protein expression using lentivirus activation particles on AML blast and progenitor survival. **a** Effect of increased SOX9 expression on oil Red O staining as compared with control AML-MSCs. Quantification of oil Red O expression between AML-MSCs induced to adipogenesis vs. AML-MSCs induced to adipogenesis after SOX9 activation (*p = < 0.05; *n* = 4). **b** Effect of MSC SOX9 expression on cell number (upper panel, *p = 0.0194, ns = not significant; *n* = 3) and CFU-L outgrowth (lower panel, *p ≤ 0.0001, **p = 0.0053, ns = not significant; *n* = 3)

As shown above, the RNA-Seq and western blot results indicated a decrease in *EGR2* and *SOX9* gene and protein, respectively in AML-MSCs. Because SOX9 affects both CEBP (CCAAT/enhancer-binding protein)β and CEBPσ, we focused whether its overexpression in AML-MSCs could affect lipid production during induction as measured by oil Red O staining. The *SOX9* gene was overexpressed using lentiviral activation particles. Oil Red O quantification showed that control AML-MSCs induced to adipogenesis had a higher percentage of oil Red O staining than did those of AML-MSCs induced to adipogenesis after SOX9 activation (Fig. 7a). This was also demonstrated to a lesser extent in ND-MSCs where baseline expression of SOX9 is higher and baseline expression of oil Red O is often <10% (data not shown). Furthermore, when both ND-MSCs and AML-MSCs were induced to chondrogenesis, the activation of SOX9 increased the maximal area of the cell pellet stained with Alcian Blue (Supplementary Fig. 5A, B), indicating enhanced chondrogenic potential.

To investigate the contribution of SOX9 upregulation on the support of AML blast cells, AML-MSCs transduced with SOX9 activation lentivirus or control particles were induced in adipogenic media. AML blast cells from the same patient sample were added to three distinct AML-MSCs for 3 days in maintenance media after adipocyte induction. Cell viability was determined by staining with DAPI and apoptosis by Annexin flow cytometry analysis. A representative flow cytometry analysis is shown in (Supplementary Fig. 6A). The total number and percent viable AML blasts cultured alone was decreased as compared with cell number and viability of AML blasts cultured on control AML-MSC feeder layers (Fig. 7b top panel and Supplementary Fig. 6B). However, there was no difference in percent viability of AML blasts exposed to SOX9 activated MSCs and control AML-MSCs (Supplementary Fig. 6B). To determine the contribution of apoptosis to AML blast cell survival in co-culture experiments, Annexin V flow cytometry analysis was undertaken. No difference was observed among groups in the percentage of late apoptotic cells (Supplementary Fig. 6B). To see if there was a difference in apoptosis induction at earlier time points, AML blasts were added to MSCs (SOX9 activated and control) for 24 h in maintenance media for adipocyte induction. The percentage of late apoptotic cells was higher in AML blasts cultured over plastic, but SOX9 activation did not affect this (Supplementary Fig. 6C).

To document whether there was differential ability between AML-MSCs with or without SOX9 upregulation to support clonogenic progenitors, a CFU-L assay was performed. Three days of AML blast co-culture on control AML-MSCs supported higher numbers of CFU-Ls per number of cells plated as compared with AML blasts alone or from AML-MSC feeder layers with increased SOX9 expression via lentiviral activation particles (Fig. 7b, lower panel).

## Discussion

In AML, MSCs provide protection from spontaneous and drug-induced apoptosis [5, 35]. It is still unclear whether primary alterations in the marrow environment contribute to leukemogenesis or whether the environment itself is secondarily altered by the presence of clonal leukemia cells. In addition to cell–cell mediated effects, soluble mediator cross talk can alter gene expression in MSCs [36]. Marrow-derived MSCs from AML patients may have altered exosome micro-RNA profiles [37] and mitochondrial transfer may also occur between MSCs and leukemia blasts [38]. Each of these modes of cellular or soluble mediator communication could alter MSCs in AML.

Differences in MSCs isolated from leukemia patients as compared with their normal counterparts were noted here and have also been reported previously. These have highlighted differences in morphology [7], growth rate [27, 39], altered osteogenic or adipogenic differentiation capacity [14, 27, 29, 40], altered methylation signatures [27], and altered ability to support normal hematopoietic stem and progenitor cells [7, 27]. Some have found altered senescence [41] whereas others have not [39]. Other groups have also shown that at earlier passages, AML-MSCs have slower growth rates but that by passage 3, doubling times are equivalent to ND-MSCs [27].

In the work presented here, AML-MSCs demonstrated enhanced adipogenic potential. This was confirmed by oil Red O staining and by CD10 and CD92 surface expression. Furthermore, AML-MSCs demonstrated enhanced secretion of free fatty acids into conditioned medium.

Analysis of gene expression patterns comparing uninduced ND-MSCs and AML-MSCs showed differentially expressed genes that are implicated in adipogenesis. Moreover, this analysis allowed us to construct a model of adipogenic predisposition of AML-MSCs, in which SOX9 and EGR2 were underexpressed.

SOX9 contributes to the commitment of MSCs to adipogenic phenotype through negative influence on expression of CEBP-α (CCAAT enhancer-binding protein) via CEBP-β/σ [42]. Downregulation of CEBP-α is required before adipocyte differentiation can proceed [43]. When SOX9 is repressed, CEBP-α and other transcription factors involved in adipogenesis are increased. EGR2, a zinc-finger transcription factor, is a positive regulator of adipogenesis which exerts its effects via CEBP-α dependent and independent mechanisms in preadipocyte differentiation [44], although its downregulation is required for adipocyte lineage commitment, with overexpression resulting in complete blockade of adipocyte lineage commitment and differentiation [45]. In MSCs of MDS patients, WNT/β-catenin target genes such as *SOX9* have also been reported to be downregulated [46]. Herein, it was also demonstrated that activation of SOX9 diminished adipogenic induction of AML-MSCs, enhanced chondrogenic differentiation, and decreased ability of AML-MSCs to support clonogenic progenitors.

Some groups have found increased osteogenic potential in AML-MSCs whereas others have found increased adipogenic potential as noted here. AML-MSCs have been found to have increased expression of FABP4 in comparison with normal controls and delayed osteogenic potential [29]. They have also been found to have higher lipoprotein lipase expression and lower E-cadherin expression [41]. In 64 AML patients, 41 untreated, Geyh et al. [27] found reduced osteogenic differentiation potential with decreased expression of osteocalcin and osterix. In subsequent work, this group also found that inhibition of TGF-β1 could restore the osteogenic differentiation capacity of AML and MDS-derived stromal cells [47].

In contrast, Battula et al. [14] found that AML cells induced osteogenic differentiation in MSCs and inhibited adipogenic differentiation. Osteogenic markers such as RUNX-2, osterix, osteopontin, and tissue nonspecific alkaline phosphatase were increased. Boyd et al. [15] found suppression of adipocytes in AML that led to inhibition of endogenous hematopoietic stem and progenitor cells. A PPARγ agonist induced marrow adipogenesis, and this rescued normal progenitor outgrowth while suppressing leukemia growth. This group found that osteoblast numbers remained stable under AML conditions [15].

In this work where we directly compared ND-MSCs with AML-MSCs, genes involved in pre adipogenesis were altered and FABP4 expression was increased providing evidence for adipogenic propensity of AML-MSCs. As noted previously, MSC heterogeneity across AML subtypes is minimal [32, 33, 40]. Jacamo et al. [48] found that despite differences in genotype and p53 status between four syngeneic primary AMLs, BM-MSCs from these mice shared certain transcription changes. Osteoblast maturation and differentiation were inhibited as were the mineralization and apoptosis of chondrocytes. Adipogenesis factors were upregulated in three of the subtypes tested. This work suggested that heterogeneous AMLs regulated the transcriptome of MSCs in similar fashion. Lu et al. [49] found that AML marrow in remission had less adipocyte content than cases from nonremission marrows as compared with diagnostic marrows. This was mediated by GDF15 (growth differentiation factor 15) from MNCs which inhibited adipogenesis independent of AML subtype. These findings as well as the demonstration here that both AML-MSCs and ND-MSCs induced to adipogenesis had superior ability to support CFU-Ls than did noninduced counterparts point up the importance of MSC disposition to adipogenesis in AML. This finding would suggest that the preadipogenic state of AML-MSCs confers increased ability to support leukemic cell survival. The extent to which leukemic blasts reprogram marrow stroma to generate a pro-leukemia environment [28] as compared with intrinsic differences between AML and ND MSCs leading to a preadipogenic phenotype is not certain [50]. Some studies have shown ongoing MSC changes in remission [18] and others have indicated persistence of an altered phenotype through all stages of disease [51].

Adipogenic niches are involved in solid tumor marrow colonization and growth [45, 52]. Bone marrow adipocytes are able to promote tumor growth in bone via a FABP4-dependent mechanism through induction of interleukin-1β and oxidative interaction between the FABP4 and PPAR-γ pathways [53]. Likewise in AML, marrow adipocytes induce hormone sensitive lipase and activate lipolysis, which then enables transfer of fatty acids from adipocytes to AML blasts [28], thus enhancing their survival. Adipocytes have also been shown to have antiapoptotic effects on AML cells [54] with their presence resulting in increased fatty acid β-oxidation along with upregulation of PPARγ, CD36, and BCL2 genes. Inhibition of fatty acid oxidation resulted in apoptosis in monocytic AML cells co-cultured with marrow stromal cells [54]. Ye et al. [55] found that gonadal adipose tissue lipolysis was able to support leukemia stem cells, especially those expressing CD36. These findings support the idea that an adipogenic environment is advantageous to leukemia cell survival as it is to solid tumors.

In our study, we found that AML-MSCs supported normal light density marrow survival and CFU-GM and BFU-E outgrowth as well as did ND-MSCs. Whether normal progenitors are differentially supported by AML-MSCs vs. ND-MSCs remains controversial. Chandran et al. [7] found that AML-MSCs were able to induce a block in differentiation of ND CD34^+^ CD38^−^ selected cells. Genes associated with quiescence and survival of stem/progenitor cells were increased such as ANGP51, SPP1, and SDF-1α [7]. Others have shown that AML-MSCs vs. ND-MSCs had decreased ability to support normal cobblestone area forming cells [56]. Other groups found no difference between AML-MSCs and ND-MSCs to support normal hematopoiesis in vitro [57]. Likewise, in this work, it was shown that AML-MSCs supported CFU-GMs more robustly that did ND-MSCs, but differential effects on blast apoptosis and survival were not demonstrated. Starting cell populations, culture conditions, and differences in cells used in readout assays could explains these divergent findings.

The studies reported upon here have several limitations. They are limited by the short co-culture duration allowed by ex vivo studies with primary AML blasts which do not survive well in culture without growth factor or stromal support. Also, ex vivo MSC cultivation could select subsets which may not be representative of all those found in marrow. Furthermore, MSCs are grown from marrow aspirates which may not extract such cells from all locations equally. While MSCs from human and murine marrow can be prospectively isolated and sorted, these cells are quite limited in number (estimated at 0.001–0.01% of marrow nucleated cells [58]), and while operative signaling pathways can be analysed by mass cytometry or similar means, functional assays to date have required ex vivo outgrowth, thus removing these cells from their complex in vivo environments. Also, the in vitro studies described here do not elucidate the intrinsic or acquired nature of the adipogenic changes which occur in these cells in a leukemia environment, and it is also uncertain whether the changes are reversible in vivo during remission [59]. Some of these changes do appear to be programmable by AML cell exposure [18, 41] but the alterations do appear to persist in vitro through several passages as shown here and by others [60].

Our studies have corroborated differences in human marrow-derived MSCs between normal and leukemia subjects and have shown differential expression of genes involved in adipogenesis. Adipocytes support leukemia survival by regulating their metabolic energy, and disruption of fatty acid oxidation in marrow adipocytes may be a therapeutic strategy [54]. Modulation of the *SOX9* gene was shown here to affect adipogenic potential and resultant support of leukemia progenitor cells. Enhanced marrow MSC adipogenic potential could lead to a pro-tumoral microenvironment and its modulation may therefore represent a novel means to inhibit AML.

## Supplementary information

Revised Supplementary Information Clean
