# Supplementary material for: Bone marrow mesenchymal stromal cells from acute myelogenous leukemia patients demonstrate adipogenic differentiation propensity with implications for leukemia cell support
Source: Leukemia. 2019 Sep 6;34(2):391–403. doi: 10.1038/s41375-019-0568-8 (PMC7214245; doi:10.1038/s41375-019-0568-8)
Supplement: Supplementary file 1 — Revised Supplementary Information Clean [file 41375_2019_568_MOESM1_ESM.docx]

**Supplementary Materials**

**Bone Marrow Mesenchymal Stromal Cells from Acute Myelogenous Leukemia Patients Demonstrate Adipogenic Differentiation Propensity with Implications for Leukemia Cell Support**

**Supplementary Materials and Methods**

**Isolation and culture of human MSCs**

Marrow aspirate material was diluted with 1X phosphate buffered saline (PBS Mediatech, Inc., Manssas, VA, USA), layered with a Ficoll-Paque^TM^ (GE Healthcare Bio-Sciences AB, Pittsburgh, PA, USA) gradient and centrifuged at 1600 rpm at room temperature (RT) for 30 minutes. The light density bone marrow cells (LDBM) at the interface were collected and washed in 1X PBS and counted with a hemacytometer. The LDBM cells were cultured at 5x10^5^ per cm^2^ in a tissue culture flask containing αMEM with L-glutamine (Gibco by Life Technologies, Grand Island, NY, USA) plus 10% heat inactivated fetal bovine serum (Gemini Bio-Products, West Sacramento, CA, USA) and 100 U/ml penicillin-streptomycin (Gibco) in a 5% CO_2_ incubator at 37ºC. Cultures were demipopulated after 72 hours. Full media changes were performed every 3-4 days after visually confirming presence of attached cells. Non-adherent cells were removed, and attached cells passaged after approximately 95% confluence was attained. For passaging, cells were washed once with 1X PBS, detached with 0.25% (wt/vol) trypsin plus 0.02% (wt/vol) EDTA (Mediatech Inc.) and passaged at a 1:2 ratio. MSCs from ND and AML subjects were used at passage three or four in the subsequent experiments.

**Time to Confluence**

LDBM cells at a density of 5x10^5^/cm^2^ were plated in tissue culture flasks at passage zero for both ND and AML specimens. Full media changes were performed every 3-4 days after visually confirming presence of attached cells. Non-adherent cells were removed, and attached cells passaged after approximately 95% confluence was attained. Such cells from both ND and AML donors could be serially passaged. For passaging, cells were washed once with 1X PBS, detached with 0.25% (wt/vol) trypsin plus 0.02% (wt/vol) EDTA (Mediatech Inc.) and passaged at a 1:2 ratio. The number of days between each passage for ND-MSC and AML-MSC specimens was recorded [1].

**Antibodies and flow cytometry**

MSCs harvested by trypsin/EDTA digestion were washed twice with 1X PBS and centrifuged at 1200 rpm for 8 minutes. The cell pellet was re-suspended in 1X PBS plus aqua live/dead viability dye (Thermo Fisher Life Technologies, Pittsburgh, PA, USA) on ice for 30 minutes. Cells were washed with 1X PBS plus 1% FBS and stained in a cocktail of antibodies to CD14-FITC (Clone BA-8; Santa Cruz Biotechnology, Santa Cruz, CA, USA), CD19-FITC (Clone H1B19; BD Pharmingen™, San Jose CA, USA ), CD45-FITC (fluorescein isothiocyanate; Clone H130; BD Pharmingen™), CD56-FITC (Clone NCAM 16.2; BD Pharmingen™), Alexa 700 conjugated anti-human CD44 (Clone G44-26; BD Pharmingen™), CD73 Brilliant Violet 421 (Clone AD2; BD Biosciences, San Jose, CA, USA), CD90-APC (allophycocyanin; Clone 5E10; BioLegend, San Diego, CA, USA), and CD117 Brilliant Violet 605 (Clone 104D2; BioLegend) for 30 minutes at 4°C.

MSCs were also stained with an antibody cocktail consisting of CD106-APC (Clone STA; BD Pharmingen™), CD146-PE (CloneP1H12; BD Pharmingen™), CD271 FITC (Clone ME20.4; BD Pharmingen™), CD45 PercpCy5.5 (CloneHI30, BD Pharmingen™), and CD31 PerCPcy5.5 (Clone WM59, BD Pharmingen™). Cells were washed with 1X PBS plus 1% FBS and centrifuged at 1200 rpm for 8 minutes and re-suspended in 1X PBS plus 1% FBS for analysis using a LSRII™ flow cytometer (BD Biosciences, San Jose, CA, USA). Data were analyzed by FlowJo software (Tree Star, Ashland, OR, USA) and collected on linear scale for forward and side scatter (*i.e.*, FSC and SSC, respectively) and on a log scale for fluorescence channels. The gating strategy utilized first identified the cell population of interest using forward scatter *vs.* side scatter plots. Doublets were then excluded by sub-gating on FSC-area *vs.* height and SSC-area *vs.* height. The population of MSCs was identified by using the Live/Dead staining reagent and negative expression for CD14, CD19, CD45 and CD56 FITC. A minimum of 10,000 cells was included in analysis of each sample. Negative control staining was assayed by fluorescence minus one (FMO). ND-MSCs and AML-MSCs induced to adipocytes stained to evaluate expression of CD10 PE (Clone H110a; BioLegend) and CD92-Alexa Fluor 647 (Clone VIM15; BD Pharmingen™).

**MSC clonogenic potential assay**

The ability to form fibroblast clones from LDBM was tested by the fibroblast colony forming unit (CFU-F) assay in both ND-MSCs and AML-MSCs. One million LDBM cells were seeded in six well tissue culture plates and growth medium changed every 4 days. CFU-Fs were scored macroscopically after fourteen days.

**Senescence associated (SA) β-galactosidase assay**

SA-β-gal was detected with a senescence fluorescence cell staining kit (Abcam, Cambridge, MA, USA) in both AML- and ND-MSCs according to the manufacturer’s instructions. Briefly, cells were washed with 1X PBS and stained with the senescence dye for 2 hr in a 5% CO_2_ incubator at 37ºC. Cells were then washed twice with senescence kit wash buffer and detached with 0.25% (wt/vol) trypsin plus 0.02% (wt/vol) EDTA and then analyzed via flow cytometry.

**Differentiation of MSCs**

**Osteogenic differentiation**

MSCs were seeded in 6 well plates coated with 1% gelatin. After cells reached 100% confluence, growth media were replaced with α-MEM supplemented with 10% FBS and 1% Pen-Strep, glutamine, 10 nM dexamethasone (Sigma-Aldrich, St. Louis, MO, USA), 100 mM L-ascorbic acid (Sigma- Aldrich) and 10 mM β-glycerophosphate (Sigma-Aldrich). Every three days, media was changed and two rounds of induction were performed. At the end of 6 days of differentiation, media were removed and cells were fixed with 10% Neutral Buffered Formalin (VWR, Radnor, PA, USA) for 40 minutes. These cultures were stained with 0.2% Alizarin Red S for 30 minutes (Sigma-Aldrich) to identify calcium deposition. Images were taken with a light microscope (Olympus America, Inc., Melville, NY, USA). At least 5 light phase contrast images were obtained from each well in triplicate using an Olympus CKX411 inverted microscope and camera. Quantification of mineralization was performed according to an established protocol [2]. Briefly, 10% glacial acetic acid (VWR) was added to each well and incubated at room temperature (RT) in a

shaking plate for 30 minutes. Cell monolayers were removed and overlaid with mineral oil (Remel, Lemexa, KS, USA) and heated to 85ºC for 10 minutes. Samples were then placed on ice for 5 minutes and then centrifuged at 20,000g for 15 minutes. Five hundred μl of supernatant was placed in a microcentrifuge tube and 200 μl of 10% (v/v) ammonium hydroxide (VWR) was added to vials. For OD measurements, 150 μl of mixture was placed in a 96 well plate in triplicate wells per sample. Optical observance measures at 405 nm were made using a plate reader (Synergy™ 2; BioTek^®^ Instruments, Inc. Winooski, VT, USA).

**Adipocyte induction**

After MSCs reached 100% confluence in 6 well tissue culture plates, fat induction medium containing α-MEM supplemented with 10% FBS and 1% Pen-Strep, glutamine, rosiglitazone (10μM; Sigma-Aldrich), dexamethasone (1μM; Sigma-Aldrich), isobutyl-1-methylxanthine (IBMX; 25μM; Cayman Chemical; Ann Arbor, Michigan, USA) and insulin (0.2unit/ml; Humulin^®^ R; Lilly LLC; Indianapolis, IN, USA) were added**.** Induction media were changed after three days and replaced with maintenance media containing insulin (0.2 unit/ml) for 24 hr. This cycle of three days induction and one-day maintenance was repeated two times. At the end of round two, maintenance media were added to cells for 6 days. Following a total of 14 days of adipocyte induction, the medium was removed and cells were washed with 1X PBS and fixed with 10% Neutral Buffered Formalin for 45 minutes. After removal of fixative, cells were stained with freshly diluted and filtered oil Red O solution (Electron Microscope Sciences, Hatfield, PA, USA) for 30 minutes. Cells were washed with 1x PBS and images taken with a light microscope (Olympus America) equipped with a camera (Olympus Inc.). Positive staining with oil Red O as an indicator of intracellular lipid accumulation determined the presence of adipocytes. At least 5 light phase contrast images were obtained from each well in triplicate using Olympus CKX411 inverted microscope and camera. The percentage of MSCs positive for oil Red O was determined by a blinded observer, and the percentages were averaged [3]. CD10 and CD92 have been reported as novel markers of adipogenic differentiation in human MSCs [4]. The surface marker expression of CD10 and CD92 was determined from ND- and AML-MSCs after adipocyte differentiation.

**Chondrocyte induction**

Culture-expended ND -and AML-MSCs were trypsinized, washed with complete culture media and then centrifuged at 1200 rpm for 8 min. The cell pellet was placed in MesenCult^TM^-ACF chondrogenic differentiation medium plus supplement from StemCell Technology following manufacturer’s protocol. Media were changed every three days for a total of 21 days of chondrogenic differentiation. Cell pellets were fixed in 10% Neutral Buffered Formalin for one hour at room temperature. Fixative was removed and 500 μl of warm Histogel (ThermoFisher Scientific) was added to each tube with a cell pellet. The tube was placed on ice for 30 minutes. The cell pellet was processed and embedded in paraffin. Consecutive sections were cut from every 5 μm and mounted onto glass slides. Alcian-Blue (EMD Millipore corporation) staining for cartilage was performed on samples [5]. Quantification of chondrocytic cell pellet area was performed with Image J software (NIH, Bethesda, MD, USA).

**Conditioned medium (C.M.) from MSCs**

Growth medium was removed from confluent tissue culture flasks of AML-MSCs and ND-MSCs. Flasks were gently washed three times with 1X PBS. α-MEM growth media without 10% FBS was added to ND-MSC and AML-MSC culture flasks. After 24 hours, medium was collected and spun at 4000 rpm to remove any residual cells. Supernatant was filtered to remove residual cells remaining. Supernatants were collected and stored at -80ºC.

**Measurement of free fatty acid**

Free fatty acid (FFA) levels in conditioned media without 10% FBS collected from AML-MSCs and ND-MSCs were determined by Zenbio (Research Triangle Park, NC, USA) ELISA assay kit per manufacturer’s instructions.

**RNA extraction**

Total RNA was extracted from MSCs using a QIAshredder homogenizer and the RNeasy plus Mini Kit (Qiagen, Valencia, CA, USA) following manufacturer’s protocol. RNA quality was assessed by Nanodrop 1000 (ThermoFisher Scientific).

**RNA-Seq**

RNA-Seq was performed by the Genomics Research Center of the University of Rochester. RNA integrity was determined on an Agilent Bioanalyzer (Agilent Technologies. Santa Clara, CA, USA). Approximately 100 ng total RNA was utilized per sample. Polyadenylated RNA was enriched, fragmented, and converted to cDNA with the Illumina TruSeq^TM^ sample preparation kit (Illumina, San Diego, CA, USA). Sequencing of the cDNA (RNA-Seq) was performed to a depth of more than 20 million reads (100-nt reads per sample) with the Illumina HiSeq2500v4. Read quality filtering was performed using Trimmomatic-0.32 with the following parameters: "SLIDINGWINDOW:4:20 TRAILING:13 LEADING:13 ILLUMINACLIP: adapters. fasta:2:30:10 MINLEN:15". Cleaned read data for each sample was aligned to the human genome (hg38) with STAR-2.5.2b and gene-level abundance measures and differential expression analysis was performed using Cufflinks-2.0.2 [6] with these parameters: "--library-type fr-firststrand --FDR 0.05 -u -b GENOME". The R package, pheatmap_1.0.8 was used in an R 3.4.1 environment to create the hierarchically clustered heat map showing raw-scaled log(fpkm+1) expression values.

**Gene ontology and pathway analysis**

Differentially expressed genes were further analyzed to find biological functions and canonical pathways with over-representation of these genes through use of Ingenuity Pathway Analysis software (IPA; Qiagen Bioinformatics (Redwood City, CA, USA). IPA aggregate data from numerous public warehouses of gene functions and interactions, as well as direct and indirect relationships curated from the scientific literature were used to test for enrichment of genes from a user-determined list. p values for enrichment are calculated to determine the statistical significance of over-representation of genes related to particular functions or pathways in the data set [7].

**Western blotting**

This was performed as previously described [8]. Briefly*,* MSCs were trypsinized as described above, washed three times with 1X PBS and centrifuged at 1200 rpm for 8 minutes. Cell pellets were lysed with M-PER mammalian protein extraction reagent (Thermo Fisher Scientific) containing 1% Protease inhibitor cocktail (Thermo Fisher Scientific). Lysates were centrifuged at 10,000 rpm for 20 minutes, the supernatant was collected and protein concentration was determined using Pierce™ BCA protein assay kit (Thermo Fisher Scientific). Proteins were separated on a 4-20% Tris-Glycine gel (Bio-Rad Laboratories, Inc., Hercules, CA, USA) and transferred to polyvinylidene membranes (PVDF; Immobilon® -FL; ThermoFisher Scientific). Membranes were probed with primary antibodies to Sox9 and Egr-2 (1:500) and GAPDH (1:1000) from Cell Signaling Technologies (Danvers, MA, USA). After washing and incubating with secondary antibody, immunoreactive proteins were visualized by using the SuperSignal™ West Femto (Thermo Fisher Scientific) detection system. Quantification analysis of Western blot bands was performed with Image J software.

**Quantitative PCR (qPCR) analysis**

cDNA from ND-MSCs or AML-MSCs was used for qPCR. qPCR was performed using TaqMan^®^ Gene Expression Master Mix (Applied Biosystems/ Thermo Fisher Scientific) and the QuantStudio 12K Flex Real-Time PCR System (Thermo Fisher Scientific). Taqman Assay Primer/probe sets for human EGR2 (Hs00166165_m1), human SOX9 (Hs01001343_g1), human FABP4 (α2P; Hs01086177_m1), human PPARγ (Hs01115513_m1) and human 18S ribosomal RNA (Hs03928990_g1) were purchased from Thermo Fisher Scientific. Human 18S rRNA was used as an endogenous control. Data were analyzed using the 2^-∆∁t^ method after normalization to 18S rRNA. Three samples of each group and 3 replicates each time were detected.

**Cell viability and apoptosis**

Percent live cells and percent apoptotic cells were assessed using 4’, 6-diamidino-2-phenylindole (DAPI; BioLegend) and a BD Pharmingen™ Annexin V APC Kit according to the manufacturer's instructions.

**MSC co-culture to assess Leukemia colony forming units (CFU-Ls**), **granulocyte-monocyte colony forming units (CFU-GMS) and burst-forming unit-erythroid (BFU-E)**

CFU-Ls were assayed as previously described [9]. Cryopreserved primary AML blasts from apheresis products were co-cultured in αMEM with L-glutamine (Gibco) with 100U/ml penicillin-streptomycin (Gibco) without serum for 72 hours with confluent layers of ND-MSCs or AML-MSCs or with ND-MSCs or AML-MSCs previously induced in adipogenic conditions. The co-cultured cells were harvested with vigorous pipetting 10 times and were plated in H4435 Enriched MethoCult™ (Stem Cell Technologies, Vancouver, BC, Canada). After a 14-day incubation at 37ºC and 5% CO_2_, CFU-Ls were scored with colonies defined as a group of greater than 20 cells. Likewise, LDBM from near age-matched normal subjects were co-cultured with confluent AML-MSCs for 72 hours in αMEM with L-glutamine (Gibco) with 100U/ml penicillin-streptomycin (Gibco) with 10% heat inactivated fetal bovine serum (Gemini Bio-Products). Cells were then collected and plated in standard CFU-GM or BFU-E assays. After a 14-day incubation at 37ºC and 5% CO_2,_ CFU-GM and BFU-E were scored, with a colony defined as a group of greater than 20 cells. In other experiments, co-culture was conducted in serum free medium for 24 hours and cell count/viability and Annexin V expression determined. To document that both the adherent and non-adherent AML cells were harvested from monolayers, after standard pipetting to remove co-cultured cells, monolayers were trypsinized and analysed by flow cytometry for CD45 expression. The mean percentage of CD45 cells was 2.07 ± 1.91 (SEM); n=6.

**Supplementary Figures and Legends**

**Supplementary Figure 1: Surface antigen expression, *in vitro* growth rate, apoptosis rate and percent senescence of bone marrow derived mesenchymal stem cells.** **(A1)** Plot of percentage surface antigen expression from ND and AML-MSCs (p=0.1; n=4).

**(A2)** Gating strategy for surface antigen expression on MSCs is shown using CD73 as an example. **(B)** Percent apoptosis as detected by Annexin V expression in ND-MSCs *vs.* AML-MSCs (p=0.1; n=5). **(C)** Quantification of β-gal positive cells in ND-MSCs and AML-MSCs (p= 0.46, n=5). (D) AML-MSCs grew more slowly during early passages as compared to ND-MSCs and the differences were statistically significant at passage 1 and 2 (*p=0.0058 and **p=0.00013; n = 6) but this difference was not observed at p3 and in later passages (data not shown).

**
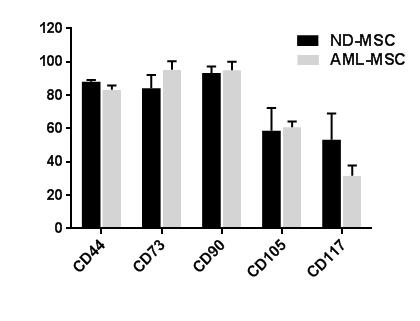
**

**A1**

**Surface Antigen**

**Expression (%)**

**A2**


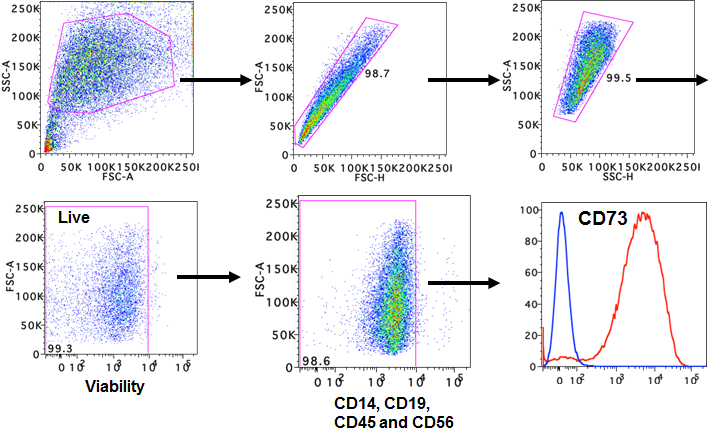


**B**


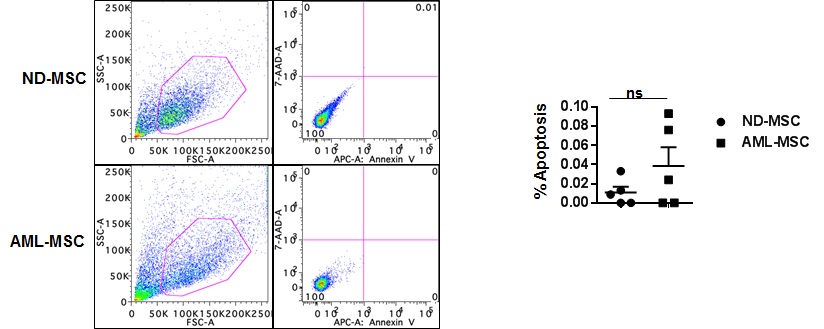


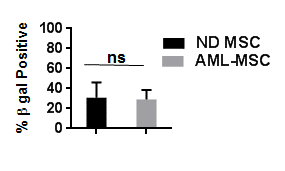


**C**

**D**

**Days**

**Passage**

**Supplementary Figure 2:** **Pathway analysis of AML- and ND-MSCs downregulated genes.** Canonical pathways were assigned by IPA where increasing value of –log(p value) indicates increasing confidence for the pathway. Pathways with values above the red threshold line (gray bars) are considered significantly affected.


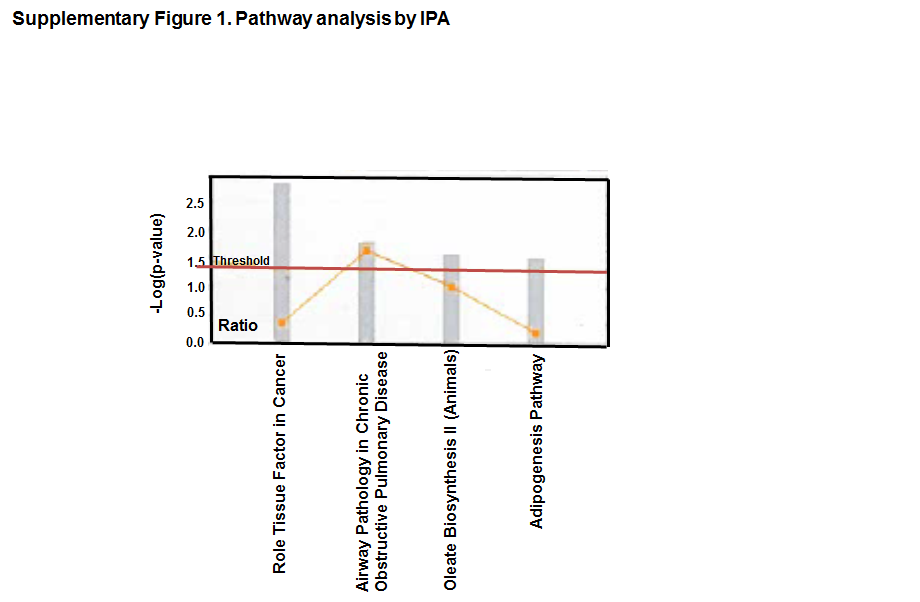


**Supplementary Figure 3:** **qRT-PCR of FABP4 (αP2) and PPARγ expression.** Reduced expression of FABP4 was shown by qRT-PCR in ND-MSCs as compared to AML-MSCs (p=<0.05; n=3). No difference in PPARγ expression was detected between groups by qRT-PCR.


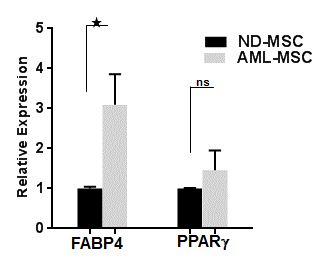


**Supplementary Figure 4**: **Annexin V expression and percentage live AML blasts co-cultured on AML-MSCs and ND-MSCs induced and non-induced in adipogenic media.** (A) Representative flow cytometry plots of AML blasts stained with Annexin V and DAPI (4’, 6-diamidino-2-phenylindole) after plastic, ND-, or AML-MSC co-culture for 72 hours. (B) Top panel. The percentage of live (*p=0.0164, **p=0.0058, ns=not significant; n=3) or late apoptotic AML blasts (*p=0.0447, **p=0.009, ns=not significant; n=3) cultured over AML-MSCs induced to adipocytes or uninduced as compared to blasts cultured in a plastic culture dish for 24 hours. (B) Bottom panel. Percentage live (ns=not significant; n=3) or late apoptotic AML blasts (*p=0.0204, **p=0.0343; n=3) cultured on ND-MSCs induced to adipocytes or uninduced as compared to blasts cultured over plastic for 24 hours.
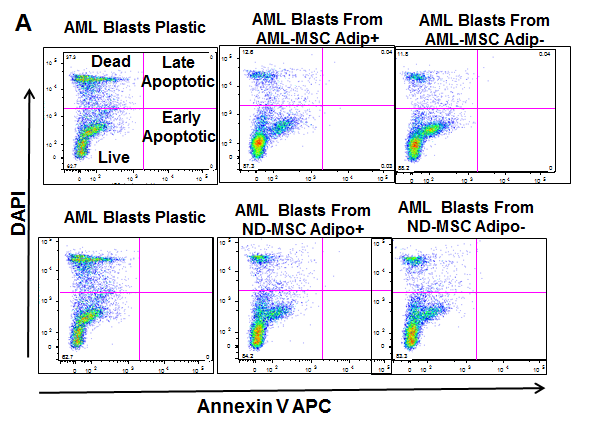


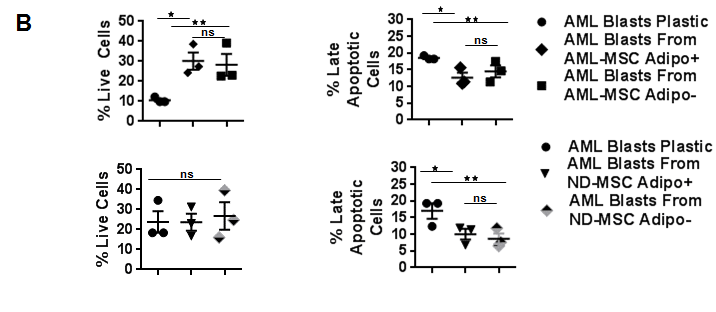


**Supplementary Figure 5: Chondrogenic differentiation of control and SOX9 activated MSCs.** (A) Effect of enhancement of SOX9 protein expression using lentivirus activation particles on chondrogenic differentiation of ND-MSCs and AML-MSCs. Enhancement of SOX9 protein in ND-MSCs and AML-MSCs resulted in larger cell pellet formation as compared to control (*p=0.0007,**p=0.0022); n=3). B) A representative comparison of Alcian-blue stained pellets between control cultture (left) and SOX9 activated culture (right).


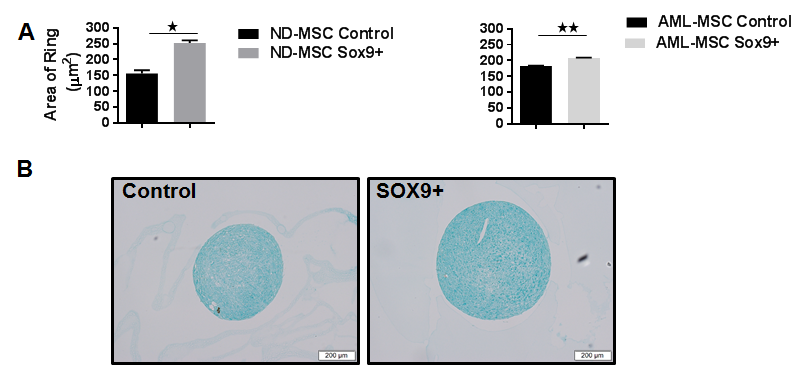


**Supplementary Figure 6: Effect of enhancement of SOX9 protein expression using lentivirus activation particles on AML blast survival.** (A) Representative flow cytometry plots of AML blasts stained with Annexin V and DAPI in co-culture conditions as specified. (B) Effect of SOX9 activation on percentage live (ns=not significant; (*p=<0.05; n=3) or late apoptotic AML blasts cultured on AML-MSC as compared to blasts cultured over plastic for 72 hours. (C) Top panel. Effect of SOX9 activation on percentage live (*p=0.0043, **p=0.0.0137, ns=not significant; n=3) or late apoptotic AML blasts (*p=0.0066, **p=0.0062, ns=not significant; n=3) cultured on ND-MSCs. (C) Bottom panel. Effect of SOX9 activation on % live (*p=0.0417, ns=not significant; n=3) or late apoptotic AML blasts (*p=0.0335, **p=0.0080, ns=not significant; n=3) cultured on AML-MSCs for 24 hours.


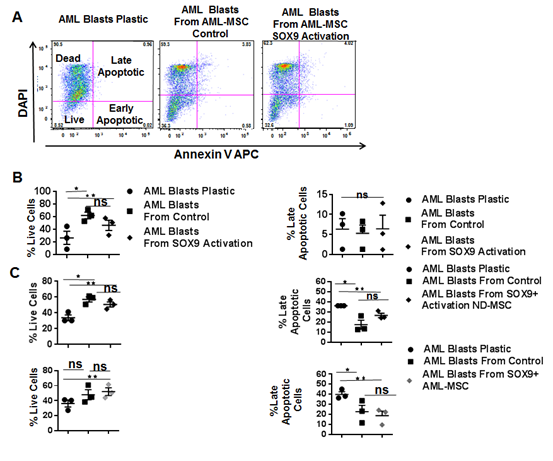


**Supplementary Table 1:** Characteristics of AML and normal donor (ND) samples. Age in years; M=Male and F=Female; FAB= (French American British); WBC= white blood count per μl; FLT3=*fms*-like tyrosine kinase 3; NPM1=nucleophosmin 1; N=negative; U=unmutated, ITD=internal tandem duplication; TKD=tyrosine kinase domain. The asterisk refers to a lymphoma sample with no bone marrow alteration.


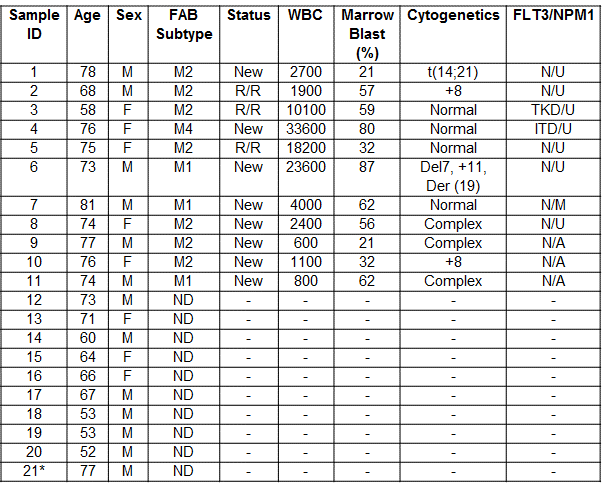


**Supplementary Table 2:**  List of upregulated genes from RNA-Seq analysis.


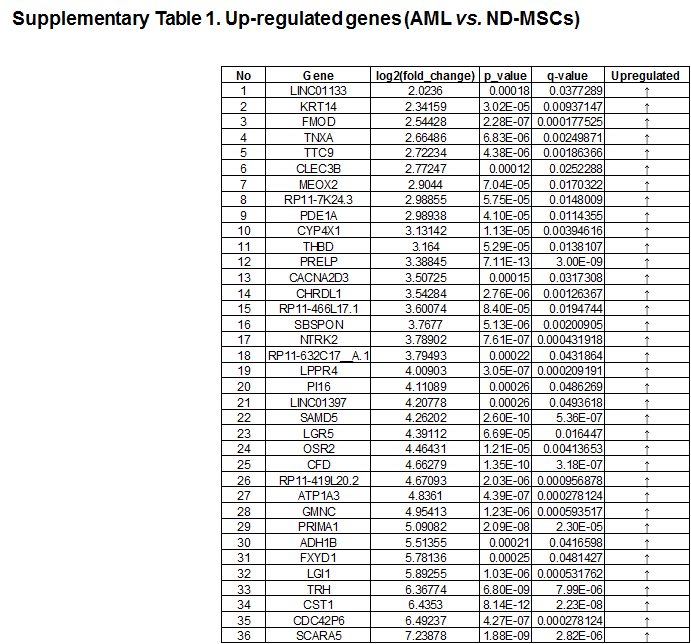


**Supplementary Table 3:** List of downregulated genes from RNA-Seq analysis.


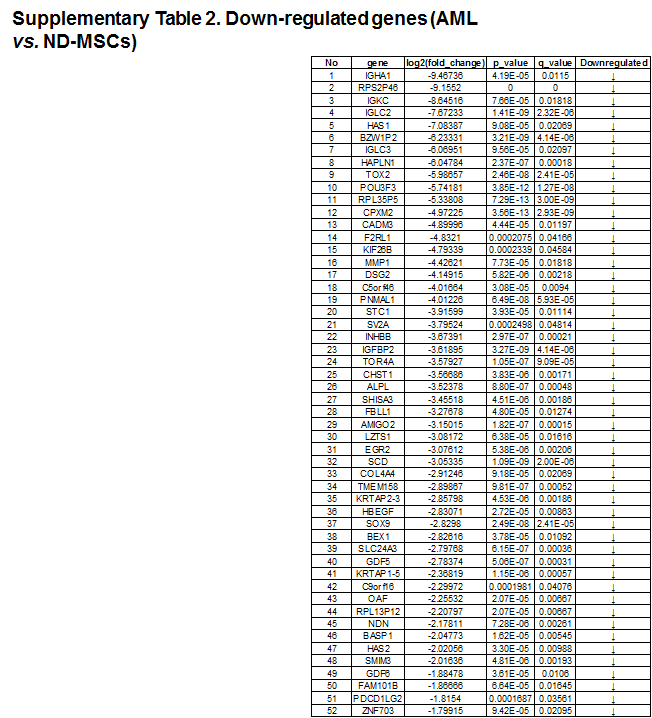


**References**

1. Pizzo RJ, Azadniv M, Guo N, Acklin J, Lacagnina K, Coppage M*, et al.* Phenotypic, genotypic, and functional characterization of normal and acute myeloid leukemia-derived marrow endothelial cells. *Exp Hematol* 2016 May; **44**(5)**:** 378-389.

2. Gregory CA, Gunn WG, Peister A, Prockop DJ. An Alizarin red-based assay of mineralization by adherent cells in culture: comparison with cetylpyridinium chloride extraction. *Anal Biochem* 2004 Jun 1; **329**(1)**:** 77-84.

3. Shafat MS, Oellerich T, Mohr S, Robinson SD, Edwards DR, Marlein CR*, et al.* Leukemic blasts program bone marrow adipocytes to generate a protumoral microenvironment. *Blood* 2017 Mar 09; **129**(10)**:** 1320-1332.

4. Graneli C, Thorfve A, Ruetschi U, Brisby H, Thomsen P, Lindahl A*, et al.* Novel markers of osteogenic and adipogenic differentiation of human bone marrow stromal cells identified using a quantitative proteomics approach. *Stem Cell Res* 2014 Jan; **12**(1)**:** 153-165.

5. Mackay AM, Beck SC, Murphy JM, Barry FP, Chichester CO, Pittenger MF. Chondrogenic differentiation of cultured human mesenchymal stem cells from marrow. *Tissue Eng* 1998 Winter; **4**(4)**:** 415-428.

6. Trapnell C, Hendrickson DG, Sauvageau M, Goff L, Rinn JL, Pachter L. Differential analysis of gene regulation at transcript resolution with RNA-seq. *Nat Biotechnol* 2013 Jan; **31**(1)**:** 46-53.

7. Huang JC, Basu SK, Zhao X, Chien S, Fang M, Oehler VG*, et al.* Mesenchymal stromal cells derived from acute myeloid leukemia bone marrow exhibit aberrant cytogenetics and cytokine elaboration. *Blood Cancer J* 2015 Apr 10; **5:** e302.

8. Liesveld JL, Bechelli J, Rosell K, Lu C, Bridger G, Phillips G, 2nd*, et al.* Effects of AMD3100 on transmigration and survival of acute myelogenous leukemia cells. *Leuk Res* 2007 Nov; **31**(11)**:** 1553-1563.

9. Liesveld JL, Rosell KE, Lu C, Bechelli J, Phillips G, Lancet JE*, et al.* Acute myelogenous leukemia--microenvironment interactions: role of endothelial cells and proteasome inhibition. *Hematology* 2005 Dec; **10**(6)**:** 483-494.
